# Novel Insights into CKMB, Myoglobin, and Troponin I Levels as Predictors of COVID-19 Severity and Hospitalization Outcomes

**DOI:** 10.3390/biomedicines13030672

**Published:** 2025-03-10

**Authors:** Aida-Isabela Adamescu, Cătălin Tilișcan, Laurențiu Mihăiță Stratan, Nicoleta Mihai, Oana-Alexandra Ganea, Sebastian Ciobanu, Adrian Gabriel Marinescu, Victoria Aramă, Ștefan Sorin Aramă

**Affiliations:** 1 Department II, Pathophysiology and Immunology, Faculty of Dental Medicine, Carol Davila University of Medicine and Pharmacy, 020021 Bucharest, Romania; sebastian.ciobanu@drd.umfcd.ro (S.C.); sorin.arama@umfcd.ro (Ș.S.A.); 2 Prof. Dr. Matei Bals National Institute of Infectious Diseases, 021105 Bucharest, Romania; mihaita.stratan@drd.umfcd.ro (L.M.S.); oana-alexandra.ganea@drd.umfcd.ro (O.-A.G.); adrian-gabriel.marinescu@drd.umfcd.ro (A.G.M.); victoria.arama@umfcd.ro (V.A.); 3 Department II, Infectious Diseases, Faculty of Medicine, Carol Davila University of Medicine and Pharmacy, 020021 Bucharest, Romania; 4 Emergency University Hospital, 050098 Bucharest, Romania

**Keywords:** COVID-19, cardiac biomarkers, hospital length of stay (LOS), risk stratification

## Abstract

**Background**: COVID-19 has largely become an endemic disease in many regions, with sporadic outbreaks, with some areas where the disease shows a seasonal pattern like the influenza virus. The focus has shifted towards managing mild and moderate forms of disease through outpatient care, aiming to prevent healthcare system overload. Consequently, identifying markers that could be used in stratifying the risk and the prognostic assessment has become crucial. Cardiovascular implications of COVID-19 are a critical area of research due to their significant impact on disease severity, mortality, and morbidity. **Methods**: We conducted a retrospective, observational study and included 472 patients, diagnosed with COVID-19, all of whom were admitted to Prof. Dr. Matei Bals National Institute of Infectious Disease, Bucharest, Romania. Levels of cardiac biomarkers like creatine kinase (CK), creatine kinase-myocardial band (CKMB), myoglobin, troponins, and NT-pro-BNP were measured and analyzed in relation to clinical presentation and outcomes. **Results**: We combined CKMB, myoglobin, and troponin I to predict hospital length of stay (LOS). Our model significantly predicted LOS (F = 12.537, *p* = 0.0001), with higher levels associated with prolonged stays (β = 0.166, *p* = 0.000). Logistic regression demonstrated that the combination of elevated CKMB and myoglobin levels significantly increased the odds of a longer LOS (OR = 1.679, *p* = 0.000). Furthermore, we found significant correlations with acute respiratory failure (*p* = 0.001), severe forms of disease (*p* = 0.000), and the development of complications during hospitalization (*p* = 0.027). **Conclusions**: These findings emphasize the value of combining cardiac biomarkers to stratify risk and predict hospital outcomes in COVID-19 patients. Routine cardiac monitoring and targeted management strategies could decrease the risk of complications, reducing the LOS. Our findings highlight the potential of cardiac biomarkers as prognostic tools to stratify risk, guide clinical interventions, and improve outcomes in COVID-19 patients.

## 1. Introduction

Cardiovascular implications in COVID-19 are being intensively studied, as their importance stems not only from their direct impact on disease severity but also from their significant contribution to mortality and morbidity [1]. Recent research has provided insights into how SARS-CoV-2 infection affects the cardiovascular system. The virus has both direct effects on the heart and blood vessels, as well as the potential to induce complications in patients with pre-existing cardiovascular conditions, diabetes, and other risk factors [2,3].

For instance, studies have demonstrated that elevated cardiac biomarkers such as troponin, myoglobin, and CKMB correlate with worse clinical outcomes, including prolonged hospitalizations and higher mortality rates in COVID-19 patients [4,5]. These findings underscore the importance of identifying biomarkers that can predict not only the severity of the disease but also the likelihood of complications, which is the focus of our study.

The indirect mechanisms by which SARS-CoV-2 affects the cardiovascular system (CVS) involve systemic inflammation, characterized by an excessive and imbalanced secretion of pro-inflammatory and anti-inflammatory cytokines, the downregulation of angiotensin-converting enzyme 2 (ACE2) receptors, endothelial dysfunction (also called ‘endothelialitis’), and hypercoagulability, especially in severe forms of the disease [6,7,8].

Recent studies have demonstrated that the inflammation triggered by SARS-CoV-2 exacerbates cardiovascular risk factors, leading to adverse outcomes such as acute myocardial infarction, which is associated with long-term complications and worsened prognosis in COVID-19 patients [6]. Additionally, the role of ACE2 receptors in viral entry and cardiovascular complications has been well documented, emphasizing their crucial involvement in endothelial dysfunction and subsequent organ damage [7]. Furthermore, the hypercoagulability seen in COVID-19 patients significantly contributes to thrombotic events, adding to the severity of cardiovascular complications [8].

The most common CV complications in COVID-19 are myocardial injury with myocarditis, acute myocardial infarction, arrhythmias (also cardiomyopathy with re-entrant arrhythmia in post-acute infection, due to fibrosis that can be induced by inflammatory cytokines or from the viral infection itself), heart failure, pericarditis, and even Takotsubo syndrome. Nevertheless, coagulation abnormalities like deep vein thrombosis and venous thromboembolism are also among the complications [9,10,11,12,13,14].

There are specific cardiac biomarkers like creatine kinase (CK), creatine kinase-myocardial band (CKMB), B-type natriuretic peptide (BNP), N-terminal pro-BNP (NT-proBNP), cardiac troponin T(cTnT), cardiac troponin I (cTnI), suppression of tumorigenicity 2 (ST2), soluble sST2 (sST2), growth differentiation factor-15 (GDF-15), galectin-3, matrix Gla-Protein species (MGPs) and even micro-ribonucleic acids (miRNAs) which are known for their roles in diagnosing, assessing, and predicting various cardiovascular conditions, being markers of myocardial injury [15]. While previous studies have focused on the diagnostic and prognostic value of cardiac biomarkers in acute cardiac events, their specific impact on complications and prolonged hospitalization in COVID-19 patients has not been fully investigated.

Myoglobin has been identified as a predictor of mortality in both severe and critical forms of COVID-19 [16]. A study from China showed that all patients with elevated troponin levels developed myocardial injury, and 50% of them died [17,18,19]. High levels of troponin were also observed in patients who required both non-invasive and invasive mechanical ventilation, as well as in those who developed ARDS (acute respiratory distress syndrome) [20,21].

Interestingly, aside from indicating myocardial injury, CK-MB has also been shown to serve as a marker of intensified immune response in patients with SARS-CoV-2 infection [19,20]. NT-proBNP, a marker of myocardial stress, along with BNP [21], should be interpreted with caution, as high values may also indicate pre-existing cardiac disease [22]. Additionally, patients with pre-existing cardiac failure and elevated biomarkers before contracting SARS-CoV-2 are considered to have a worse prognosis [23].

All three biomarkers, including CK, have been associated with higher mortality, and elevated levels of these biomarkers were identified in all deceased COVID-19 patients [21,24,25]. While myocardial injury is common among COVID-19 patients, its frequency varies based on several individual factors [26].

In our study, we demonstrated a link between increased levels of some of these biomarkers and prolonged hospital stays, the presence of acute respiratory failure, and the development of complications during hospitalization, which highlights their potential as prognostic markers.

## 2. Materials and Methods

We conducted a retrospective, observational study in which we included 472 patients admitted to Prof. Dr. Matei Bals National Institute of Infectious Diseases, Bucharest, Romania. The study was conducted between 1 January 2021 and 20 October 2021. Participants who had a positive SARS-CoV-2 rapid antigen test or PCR were included, while those who tested negative or were under 14 years old were excluded. We obtained informed consent from all patients, or in the case of those aged 14–18, from their legal representative. The study protocol was approved by the Ethics Committee of Prof. Dr. Matei Balș National Institute of Infectious Diseases, Bucharest, Romania (protocol No. C0408/2020, 20 October 2021).

Demographic data, including age and gender, as well as clinical parameters such as form of disease, signs and symptoms, comorbidities, biochemical and hematological parameters such as inflammatory biomarkers, cardiac biomarkers such as CK, CKMB, myoglobin, troponin I, NT pro-BNP, complete blood count (CBC), cholestasis enzymes (alkaline phosphatase, gamma-glutamyl transferase), and aspartate aminotransferase and alanine transaminase levels, complications, and hematological indices, were collected. All data were collected anonymously and organized in a structured database using Microsoft Excel.

Descriptive statistics such as means, medians, and frequencies were used to summarize the data. Bivariate regression analyses were conducted to examine the relationships between independent and dependent variables. Variables with a *p*-value of 0.05 in the bivariate analysis were included in a step-up multivariate logistic regression model to identify independent predictors. All statistical analyses were performed using SPSS version 20 (IBM Group, Armonk, NY, USA) guaranteeing adherence to statistical standards and reproducibility guidelines. Before analysis, data were checked for normality using the Shapiro–Wilk test. Necessary transformations were applied to ensure the validity of statistical assumptions.

## 3. Results

We included 472 anonymized patients in our study, consisting of 195 women (41.3%) and 277 men (58.7%). The median length of the hospital stay was 9.74 days (IQR: 7.34–13.33). No significant differences in hospitalization duration were observed between genders (Table 1).

In analyzing the severity, clinical presentation, and hepatic features of COVID-19 patients by gender, the results show a notable distribution across different disease forms. Of the 472 total patients, 49.8% had a severe form of COVID-19, with a higher proportion of men (55.6%) compared to women (41.5%). A moderate form of the disease was observed in 46% of patients, with women comprising 52.3% and men 41.5%. Interestingly, 4.2% of the total sample had a mild form, with women exhibiting a slightly higher rate (6.2%) than men (2.9%).

Regarding clinical symptoms, the most common was cough, reported by 86.4% of all patients, with no significant difference between genders (86.7% in women and 86.3% in men). Fatigue was also highly prevalent, affecting 64.4% of the total cohort, with nearly equal rates for women (65.6%) and men (63.5%). Other symptoms, such as myalgias, arthralgias, and gastrointestinal symptoms like diarrhea and nausea, were similarly widespread, with some variation between genders. In terms of hepatic involvement, 56.1% of all patients exhibited signs of hepatic cytolysis, with a similar distribution between women (55.9%) and men (56.3%). Cholestasis was noted in 60% of patients overall, but it was more prevalent in men (67.1%) than in women (49.7%). Additionally, 301 patients (63.8%) experienced acute respiratory failure at the time of admission. Among the 472 patients, 20 (4.2%) had a mild form of disease, 217 (46%) had a moderate form, and 235 (49.8%) had a severe form (Table 2).

A total of 236 patients (50%) developed at least one complication during hospitalization, such as bacterial superinfection. Moreover, we identified statistical correlations between acute respiratory failure and our prediction model (*p* = 0.001), the severe form of the disease (*p* < 0.000), and the development of complications during hospitalization (*p* = 0.027)

No statistical correlations were found between our model and the presence of cholestasis (*p* = 0.447), hepatic cytolysis (*p* = 0.139), ICU admission (*p* = 0.327), death (*p* = 0.748), the presence of autoimmune disease (*p* = 0.617), immunosuppression (*p* = 0.117), HIV+ (*p* = 0.94), neoplasia (*p* = 0.74), hematologic diseases (*p* = 0.658), or any other chronic conditions. Additionally, no correlations were found in patients who smoke (*p* = 0.338).

A series of biomarkers were included in the normal panel tests at admission, CK, CKMB, myoglobin, troponin I, and NT-proBNP. These biomarkers were selected due to their association with cardiac injury and inflammatory processes in COVID-19 patients. The median values of these biomarkers are listed in Table 3 and Table 4. Moreover, no statistical differences were observed between gender and the median value of our studied biomarkers (the median value of CKMB was 10 days both in men and in women; the median value of myoglobin was 100 days in women vs. 111 in men).

Multiple linear regression was conducted to assess the importance of CKMB, myoglobin, troponin I, and NT-proBNP and the duration of hospitalization (Table 5).

We first combined CKMB and myoglobin into a regression. The ANOVA showed that the combined variable significantly predicts the LOS (sum of squares regression = 395.577, df = 1, mean square regression = 395.577, F-statistic = 12.537 with a *p*-value of 0.0001). Moreover, B = 0.009, std error = 0.003, Beta = 0.166, *t*-statistic = 3.541, *p* = 0.000, and R-squared is 0.28. The positive coefficient suggests that higher levels of the combined biomarker are associated with longer hospital stays. This could indicate that the combined effect of CKMB and myoglobin is a useful marker for disease severity or prolonged recovery.

Then, we added troponin I to this model with an R-squared of 0.29, regression sum of squares = 412.930, df = 1, F = 13.048, *p* = 0.000. NT-proBNP was then added to the regression model, along with CKMB, myoglobin, and troponin I. This had a minimal impact on improving the model’s explanatory power, although it is statistically significant (*p* = 0.002).

Based on the median value of LOS, we divided the patients into two groups: those with short LOS (<9.74 days) and long LOS (>9.74 days). A bivariate logistic regression analysis was conducted to assess the relationship between combined CKMB and myoglobin levels and LOS. The results indicated a significant positive association between the combined biomarker levels and the likelihood of a longer hospital stay (B = 0.518, *p* = 0.000).

The odds ratio (Exp(B)) was 1.679, suggesting that for each unit increase in the combined biomarker level, the odds of a prolonged hospital stay increased by 67.9%.

The Omnibus test for the regression model was significant (chi-square = 9.078, df = 1, *p* = 0.003), indicating that the overall model was statistically significant. The Hosmer–Lemeshow test showed no significant difference between observed and predicted values (chi-square = 7.491, df = 8, *p* = 0.485), suggesting that the model fits the data.

A multivariable logistic regression model was then constructed by including NT-proBNP as an additional predictor. The final model showed an R^2^ of 0.29, indicating that approximately 29% of the variance in the length of hospital stay was explained by the predictors included in the model. The NT-proBNP level was found to be marginally significant (*p* = 0.002), but the effect size was small, suggesting that NT-proBNP might contribute to predicting hospital stay duration, though not as strongly as the other biomarkers.

Furthermore, we divided the patients into three groups: under 44 years old (76 patients, 16.1%), between 44 and 75 years old (327 patients, 69.3%), and over 75 years old (69 patients, 14.6%). We tested the relationship between the age group (under 44 years old) and the CKMB–myoglobin model and obtained a Pearson chi-square of 405, with df = 402, a *p*-value of 0.449, phi = 0.955, Cramér’s V of 0.955, and a contingency coefficient of 0.691.

For the second group, with patients aged 44–75, we obtained a *p*-value of 0.495, phi = 0.951, Cramér’s V of 0.951, and a contingency coefficient of 0.689. For the third group, which included patients over 75 years of age, we obtained a *p*-value of 0.328, phi = 0.966, Cramér’s V of 0.966, and a contingency coefficient of 0.695.

## 4. Discussion

SARS-CoV-2 has a diverse impact on the cardiovascular system, influenced by both individual and environmental factors [27]. Given the broad spectrum of cardiovascular conditions, it is often essential to assess multiple biomarkers to accurately evaluate the patient’s status [28]. Consistent with this, our study highlights a strong association between elevated cardiac biomarkers and adverse outcomes in hospitalized COVID-19 patients, including prolonged hospital stays, the occurrence of acute respiratory failure, and the development of complications during hospitalization. These findings are in line with previous research, which suggests that cardiac injury plays a significant role in the morbidity and mortality seen in severe COVID-19 cases [29].

Elevated CK-MB levels have been closely linked to increased disease severity and higher mortality rates in COVID-19 patients. This indicator of cardiac damage could play a valuable role in assessing risk within this population [30]. Elevated CKMB levels are associated with myocardial injury, which could complicate COVID-19 outcomes. Patients with elevated CKMB levels might experience more severe cardiovascular issues, leading to longer hospital stays. We identified CKMB as a strong predictor of how long a patient is hospitalized.

Increased levels of myoglobin are associated with increased hospital stay [31]. Similarly, our results suggest that myoglobin shows a significant positive relationship with LOS. Patients with higher myoglobin levels, which can indicate muscle damage or myocardial injury, may stay in the hospital longer. Myoglobin is often elevated in cases of muscle or cardiac injury. In the context of COVID-19, elevated myoglobin could signal more severe cardiovascular or musculoskeletal damage, which would lead to longer hospital stays due to complications, the need for additional care, or prolonged recovery.

Strong data support the association between increased cardiac troponin levels and both the intensity of disease and unfavorable outcomes in individuals with COVID-19 [32]. We discovered a negative relationship between troponin and LOS. The data suggest that higher troponin I levels (which indicate myocardial injury) might be associated with shorter hospital stays in our model.

This might seem counterintuitive, but it could reflect the severity of cardiac injury leading to early interventions or rapid deterioration that leads to quicker outcomes (death or ICU admission) in some cases. However, in general, higher troponin is more commonly associated with worse outcomes, which could explain the shorter stay for patients who experience severe cardiac injury early on [33].

CK does not show a significant relationship with the LOS. The very small B value and *p*-value suggest that CK has little to no impact on how long a patient stays in the hospital.

Since CK is a general biomarker for muscle injury, it might not be specific enough to predict hospital stay duration in COVID-19 patients, especially when compared to more targeted biomarkers like CKMB or troponin, which are directly related to cardiac events that could impact hospitalization length.

NT-proBNP does not show a significant relationship with the LOS. While NT-proBNP is a sensitive marker for heart failure and cardiac stress, it does not seem to have a strong impact on how long patients stay in the hospital in our study.

Studies have shown that an increased level of myoglobin may be caused by other pre-existing illnesses, like liver or pulmonary diseases or cancer [31]. By comparison, we did not identify any correlations between the presence of one or more comorbidities and the elevated levels of myoglobin, troponin, CK, CKMB, or NT-proBNP.

Thus, CKMB, myoglobin, and troponin I have the most significant correlations with LOS in COVID-19 patients. CKMB and myoglobin specifically show positive relationships with longer hospital stays, indicating their relevance in predicting COVID-19 severity and complications. No such correlation has been identified regarding NT-proBNP.

We can then assume that biomarkers related to cardiac injury and tissue damage are stronger predictors of hospitalization duration than more general markers. After troponin I was added to the model, R-squared only increased slightly, but the new model still holds statistical significance; thus, troponin I is a valuable contributor. Even though the model does not explain all the variance in hospital stay, it still suggests that troponin I, alongside CKMB and myoglobin, is an important predictor, and could potentially help guide clinical decisions.

Our analysis identified that combined CKMB and myoglobin levels were significant predictors of a prolonged hospital stay in COVID-19 patients. Troponin I was found to have a negative association with the length of hospital stay, while NT-proBNP showed a marginally significant relationship. These biomarkers help explain a large part of the variation in how long patients stay in the hospital, which can be helpful for managing care more effectively in clinical settings.

An American study demonstrated that all 80 patients included in the study, all of whom had elevated levels of cardiac biomarkers, required advanced respiratory support [34]. Previous research suggests that cardiac injury significantly contributes to the morbidity and mortality observed in severe COVID-19 cases, particularly in the context of respiratory failure. Troponin, myoglobin, and CK-MB levels have been identified as reliable predictors of respiratory complications, such as acute respiratory distress syndrome (ARDS), in COVID-19 patients [35].

Elevated cardiac biomarkers, particularly troponin, have been shown to correlate with poorer respiratory outcomes, with some studies even suggesting that these biomarkers could serve as early indicators of impending respiratory failure [36]. The mechanisms underlying this relationship involve myocardial injury, systemic inflammation, and vascular dysfunction, all of which are common in COVID-19. These markers may assist clinicians in predicting the onset of respiratory failure and in tailoring interventions early in the disease course [37]. Furthermore, the presence of elevated cardiac biomarkers in COVID-19 patients has been associated with higher mortality rates, especially when respiratory failure becomes a complication [38].

We have also found statistical correlations between our predictive model and various critical outcomes in COVID-19 patients like the onset of acute respiratory failure, which often requires intensive care and invasive interventions. This finding suggests that the biomarkers included in our model could serve as early indicators of respiratory failure, enabling clinicians to take preemptive measures to prevent further deterioration.

Furthermore, our model showed a strong correlation with the development of severe diseases, supporting the idea that patients with higher biomarker levels are more likely to develop severe forms of disease. Similarly to our results, studies have demonstrated that increased levels of these biomarkers are associated with the progression to more severe forms of the disease and the development of post-acute COVID-19 sequelae [31,39,40]. This is particularly important for risk stratification because it allows healthcare providers to identify high-risk patients early and apply more aggressive actions. This could not only improve outcomes but also guarantee a more efficient distribution of medical resources.

Additionally, the correlation between our model and the development of complications during hospitalization emphasizes the importance of close monitoring for patients exhibiting elevated biomarker levels. Complications such as cardiovascular events, thromboembolism, and multi-organ dysfunction are common in severe COVID-19 cases, and early identification of those at risk could lead to better-targeted interventions that minimize the impact of such complications.

We found no statistical difference between cardiac parameters for young patients and elderly patients. The *p*-value indicates no significant relationship between age (under 44) and the CKMB–myoglobin model. Despite strong values for phi, Cramér’s V, and the contingency coefficient, the lack of statistical significance means we cannot conclude that age influences the CKMB–myoglobin model in a meaningful or reliable way.

For the second group, although the association measures suggest a strong relationship, the high *p*-value indicates that this relationship is not statistically significant. Therefore, we cannot confidently conclude that CKMB–myoglobin levels are meaningfully associated with the age group of 44–75 years in our dataset.

For the third group, while the association between CKMB–myoglobin levels and the >75 age group appears strong, the *p*-value of 0.328 shows that the association is not statistically significant. This suggests the relationship could be due to chance, and there is insufficient evidence to conclude that CKMB–myoglobin levels are meaningfully associated with individuals over 75 years old.

In summary, despite the high strength of association, the lack of statistical significance (due to the high *p*-value) means we cannot conclude that CKMB–myoglobin levels reliably predict or correlate with age in this group.

These results highlight the predictive power of cardiac biomarkers and the potential of our model to guide clinical decision-making. By incorporating biomarkers into routine clinical practice, clinicians could more effectively stratify risk, prioritize resources, and improve patient outcomes. Early recognition of at-risk patients, especially those likely to experience respiratory failure, severe disease progression, and complications, could ultimately help reduce both the incidence of adverse outcomes and the burden on healthcare systems during the pandemic.

These cardiac biomarkers could be used as prognostic tools because they offer valuable information about disease severity while also shaping clinical decisions. By pinpointing patients in need of intensive monitoring or specialized treatments, these markers can optimize resource distribution, enhance patient care, and ease the strain on healthcare systems. Recognizing high-risk individuals early may help avoid complications like arrhythmia, cardiac failure, or multi-organ damage, which frequently extend hospital stays and increase treatment requirements.

Elevated levels of CKMB, myoglobin, and troponin I are strong indicators that a patient is at risk for a longer hospital stay and more severe complications. When these biomarkers are significantly higher than normal, particularly when they exceed twice the usual reference values, it suggests a higher likelihood of developing serious issues such as respiratory failure or cardiovascular events [41,42].

The practical value of monitoring these biomarkers is that they enable doctors to identify problems early, facilitating faster intervention and better patient care management. By recognizing high-risk patients early, healthcare teams can allocate resources more effectively, prevent complications, and ultimately improve patient outcomes. This approach not only enhances individual care but also ensures more efficient use of hospital resources during times of high demand.

### 4.1. Limitations of the Study

Our findings are promising, but they should be interpreted with caution due to certain limitations. Because of the study design, there are some limits to our ability to establish causality. Furthermore, our data come from a single center, which may not portray variations in patient populations or care protocols across different institutions. Nonetheless, biomarker levels could also be influenced by pre-existing cardiac conditions, which is not the focus of our research.

### 4.2. Future Directions

Although we did not specifically track the duration for which patients tested positive for COVID-19, it would be valuable for future studies to explore how the length of time a patient remains COVID-19-positive might influence disease severity and clinical outcomes. Understanding whether a longer COVID-19-positive period is associated with more severe symptoms or prolonged hospital stays could provide new insights into patient management and care strategies.

Future research should focus on how to integrate these cardiac biomarkers into the clinical management strategies for patients with COVID-19. We need a multi-center perspective to validate these findings and assess the practical and economic impact of implementing these biomarkers in everyday clinical settings. Additionally, studies could identify innovative treatment approaches.

## 5. Conclusions

Our study reveals the significant prognostic value of cardiac biomarkers in predicting adverse outcomes in hospitalized COVID-19 patients. Higher levels of biomarkers such as cardiac troponin, CK-MB, and NT-proBNP were strongly correlated with disease severity, prolonged hospital stays, acute respiratory failure, and the development of complications. These findings suggest that cardiac biomarkers can serve as valuable tools for early risk stratification and clinical decision-making, allowing healthcare providers to recognize high-risk patients who may benefit from closer monitoring and more aggressive interventions.

In clinical practice, clinicians can improve the accuracy of their prognostic calculations and optimize patient management, potentially preventing deadly complications and reducing the overall weight on healthcare systems. Future research should focus on supporting these findings through multicenter prospective studies and exploring the underlying mechanisms of cardiac injury in COVID-19 to reveal potential novel therapeutic targets that could further improve patient outcomes.

Clinicians could use these biomarkers to stratify risk and predict outcomes in COVID-19 patients. Identifying patients with elevated cardiac biomarkers could encourage closer monitoring and more aggressive interventions, potentially preventing complications and reducing overall hospital stay. These data might also stimulate research on treatments that could minimize cardiac injury, potentially improving patient outcomes and reducing the LOS. Moreover, they emphasize the importance of routine cardiac monitoring in COVID-19 patients and could guide updates to clinical guidelines, particularly for patients presenting with cardiovascular risk factors or symptoms. By using a predictive model, we could boost the clinical management of COVID-19 patients, by improving patient care and optimizing healthcare resources, especially in high-pressure clinical environments. The inclusion of cardiac biomarkers in the clinical management of COVID-19 patients holds significant promise in improving patient care and resource allocation, particularly in settings with limited healthcare resources.

## Figures and Tables

**Table 1:** Demographic characteristics, comorbidities, and clinical characteristics of COVID-19 patients by gender.

Variable	Total (n = 472)	Women (n = 195)	Men (n = 277)
Demographic Characteristics			
Age (median, IQR)	59 (47–70)	64 (52–72)	55 (45–69)
Gender, n (%)	100	41.3	58.7
Body mass index (mean, standard deviation)	28.40 (25.14–32.09)	28.80 (25.72–32.14)	28.39 (25.72–32.14)
Comorbidities, n (%)	320 (67.8)	146 (74.9)	174 (62.8)
Arterial hypertension	241 (51.1)	114 (58.5)	127 (45.8)
Type I diabetes mellitus	24 (5.1)	7 (3.6)	17 (6.1)
Type II diabetes mellitus	71 (15)	35 (17.9)	36 (13)
Chronic respiratory diseases	12 (2.5)	3 (1.5)	9 (3.2)
Chronic kidney disease	25 (5.3)	5 (2.6)	20 (7.2)
Active neoplasia	22 (4.7)	12 (6.2)	10 (3.6)
HIV	1 (0.2)	0	1 (0.4)
Length of hospital stay (median, IQR)	10 (7–13)	10 (8–13)	9 (7–13)
Smoking status *	10 (2.1)	2 (1)	8 (2.9)
Vaccination status	47 (10)	24 (12.3)	23 (8.3)

* Active smokers. Abbreviations: HIV, human immunodeficiency virus.

**Table 2:** Severity form of disease, clinical presentation, and hepatic features of COVID-19 patients by gender.

	Total (n = 472)	Women (n = 195)	Men (n = 277)
Form of disease			
Mild	20 (4.2)	12 (6.2)	8 (2.9)
Moderate	217 (46)	102 (52.3)	115 (41.5)
Severe	235 (49.8)	81 (41.5)	154 (55.6)
Signs and symptoms			
Cough	408 (86.4)	169 (86.7)	239 (86.3)
Anosmia	80 (16.9)	39 (20)	41 (14.8)
Ageusia	57 (12.1)	28 (14.4)	29 (10.5)
Fatigue	304 (64.4)	128 (65.6)	176 (63.5)
Myalgias	212 (44.9)	93 (47.7)	119 (43)
Arthralgias	112 (23.7)	50 (25.6)	62 (22.4)
Diarrhea	114 (24.2)	51 (26.2)	63 (22.7)
Nausea	109 (23.1)	52 (26.7)	57 (20.6)
Vomiting	43 (9.1)	25 (12.8)	18 (6.5)
Cephalgia	175 (37.1)	73 (37.4)	102 (36.8)
Dyspnea	210 (44.5)	79 (40.5)	131 (47.3)
Hepatic cytolysis	265 (56.1)	109 (55.9)	156 (56.3)
Cholestasis	283 (60)	97 (49.7)	186 (67.1)

**Table 3:** Levels of cardiac biomarkers in COVID-19 patients: median and interquartile range (IQR).

Elevated Levels of					
	CK	CKMB	Myoglobin	Troponin I	NT-proBNP
n = number of patients	134	113	247	34	139
%	28.4	23.9	52.3	7.2	29.4

Abbreviations: CK, creatine kinase; CK-MB, creatine kinase-MB (muscle, brain); NT-pro BNP, N-terminal pro-B-type natriuretic peptide.

**Table 4:** Frequency of elevated levels of cardiac biomarkers in COVID-19 patients.

	Median	IQR (25–75)	Reference Values
CK (U/L)	92	52–190	55–170 U/L
CK-MB (U/L)	10	7–15	1–16 U/L
Myoglobin (ng/mL)	106	70–167	0–99.3 ng/ml
Troponin I (ng/mL)	0.3	0.2–0.5	0–0.16 ng/ml
NT-proBNP (ng/L)	40	12.08–172	0–125 ng/L

**Table 5:** Association between biomarker levels and duration of hospitalization.

	CK	CKMB	Myoglobin	Troponin I	NT-proBNP
B	2.307 × 10^−005^	0.0020	0.010	−1.67	0.000
Std error	0.002	0.037	0.003	0.769	0.000
Beta	0.001	0.00322	0.171	−0.117	0.044
t	0.012	0.534	3.158	−2.171	0.808
p	0.990	0.000	0.002	0.031	0.419

Abbreviations: B, unstandardized coefficient error; Std error, standard error; Beta, standardized coefficient.

## Data Availability

The original contributions presented in this study are included in the article. Further inquiries can be directed to the corresponding authors.
